# Timeliness of contact tracing among flight passengers during the COVID-19 epidemic in Vietnam

**DOI:** 10.1186/s12879-021-06067-x

**Published:** 2021-04-28

**Authors:** Thai Quang Pham, Ngoc-Anh Hoang, Ha-Linh Quach, Khanh Cong Nguyen, Samantha Colquhoun, Stephen Lambert, Luong Huy Duong, Quang Dai Tran, Duc Anh Ha, Dinh Cong Phung, Nghia Duy Ngu, Tu Anh Tran, Quang Ngoc La, Tai Trong Nguyen, Quynh Mai Thi Le, Duong Nhu Tran, Florian Vogt, Duc-Anh Dang

**Affiliations:** 1 Department of Communicable Diseases Control and Prevention, National Institute of Hygiene and Epidemiology, Hanoi, Vietnam; 2 Institute of Preventive Medicine and Public Health, Hanoi Medical University, Hanoi, Vietnam; 3 National Centre for Epidemiology and Population Health, Research School of Population Health, College of Health and Medicine, Australian National University, Canberra, ACT Australia; 4 Medical Services Administration, Ministry of Health, Hanoi, Vietnam; 5 General Department of Preventive Medicine, Ministry of Health, Hanoi, Vietnam; 6 Ministry Office, Ministry of Health, Hanoi, Vietnam; 7 National Agency for Science and Technology Information, Ministry of Science and Technology, Hanoi, Vietnam; 8 Hanoi University of Public Health, Hanoi, Vietnam; 9 The Kirby Institute, University of New South Wales, Sydney, NSW Australia

**Keywords:** Timeliness, Contact tracing, COVID-19, SARS-CoV-2, Vietnam

## Abstract

**Background:**

International air travel plays an important role in the global spread of SARS-CoV-2, and tracing of close contacts is an integral part of the public health response to COVID-19. We aimed to assess the timeliness of contact tracing among airline passengers arriving in Vietnam on flights containing COVID-19 cases and investigated factors associated with timeliness of contact tracing.

**Methods:**

We included data from 2228 passengers on 22 incoming flights between 2 and 19 March 2020. Contact tracing duration was assessed separately for the time between the date of index case confirmation and date of contact tracing initiation (interval I), and the date of contact tracing initiation and completion (interval II). We used log-rank tests and multivariable Poisson regression models to identify factors associated with timeliness.

**Results:**

The median duration of interval I and interval II was one (IQR: 1–2) and 3 days (IQR: 2–5), respectively. The contact tracing duration was shorter for passengers from flights where the index case was identified through mandatory testing directly upon arrival (median = 4; IQR: 3–5) compared to flights with index case detection through self-presentation at health facilities after arrival (median = 7; IQR: 5–8) (*p*-value = 0.018). Cumulative hazards for successful tracing were higher for Vietnamese nationals compared to non-Vietnamese nationals (*p* < 0.001).

**Conclusions:**

Contact tracing among flight passengers in the early stage of the COVID-19 epidemic in Vietnam was timely though delays occurred on high workload days. Mandatory SARS-CoV-2 testing at arrival may reduce contact tracing duration and should be considered as an integrated screening tool for flight passengers from high-risk areas when entering low-transmission settings with limited contact tracing capacity. We recommend a standardized risk-based contact tracing approach for flight passengers during the ongoing COVID-19 epidemic.

**Supplementary Information:**

The online version contains supplementary material available at 10.1186/s12879-021-06067-x.

## Background

COVID-19 has spread globally since the beginning of 2020. Within 9 months, SARS-CoV-2 infected over 40 million people and caused over 1 million fatalities worldwide [1]. International air travel plays an important role in the global spread of COVID-19 [2]. Since the beginning of the epidemic, many countries implemented different policies to prevent the importation of COVID-19, including complete closure of borders for international travel [3], systematic passenger testing, and mandatory quarantine at arrival [4].

Vietnam has experienced many infectious disease importations during the last 20 years [5–7], however experience with flight-related contact tracing is limited. Shortly after the first COVID-19 case was reported from Wuhan, China, Vietnam’s government implemented a range of policies to screen, detect, and investigate passengers arriving on flights from COVID-19 affected regions. In March 2020, Vietnam saw a surge in COVID-19 cases among returning nationals and tourists, which subsequently triggered a series of public health measures to trace passengers from flights on which an infected passenger had been detected [8].

While contact tracing has proven critical for the containment of COVID-19 transmission in general [9, 10], there is a lack of evidence on contact tracing among aircraft passengers [11, 12]. Timeliness in particular, a key component for effective contact tracing, and factors influencing timeliness in low-middle income settings such as Vietnam, has not been investigated to date. Such evidence is crucial to improve the response to flight-related COVID-19 importations during the ongoing pandemic. We aimed to assess the timeliness of contact tracing among airline passengers arriving in Vietnam on flights with at least one confirmed COVID-19 case on board during March 2020, and explored factors associated with timeliness of contact tracing.

## Methods

### Setting

#### Passenger surveillance

In Vietnam, the Government delegated the National Steering Committee for COVID-19 Prevention and Control to implement case finding and contact tracing activities among all passengers of flights where at least one COVID-19 case was detected. This process entailed nation-wide multi-institutional cooperation to contact, test, and quarantine passengers after arrival in Vietnam.

During the course of March 2020, Vietnam’s government gradually introduced policies for compulsory SARS-CoV-2 testing and 14-day quarantine for passengers from some designed areas. This included the United Kingdom and 26 Schengen countries on 14 March [13] and was expanded to include the United States, Southeast Asian countries, Russia on 18 March [14], and expanded again to all international flights on 21 March 2020 [15]. The evolving testing policies are outlined in Additional file 1. During this period, other passengers of the same plane could leave the airport without testing or quarantine if they did not depart from such designated areas and would only be contacted when any co-passengers of their flight were confirmed to be positive for SARS-CoV-2. Consequently, during most of March 2020, flight-associated COVID-19 index cases (the first confirmed cases among flight passengers) were detected either by self-presentation or through mandatory testing in Vietnam depending on the country of departure.

For this analysis, self-presentation was defined as index cases who presented themselves at local health facilities after arrival due to COVID-19 symptoms or due to recent travel history to affected areas following public awareness campaigns. Mandatory testing was defined as index cases who were tested mandatorily for SARS-CoV-2 at immigration points directly upon arrival.

#### Contact tracing procedures

**Fig. 1:**
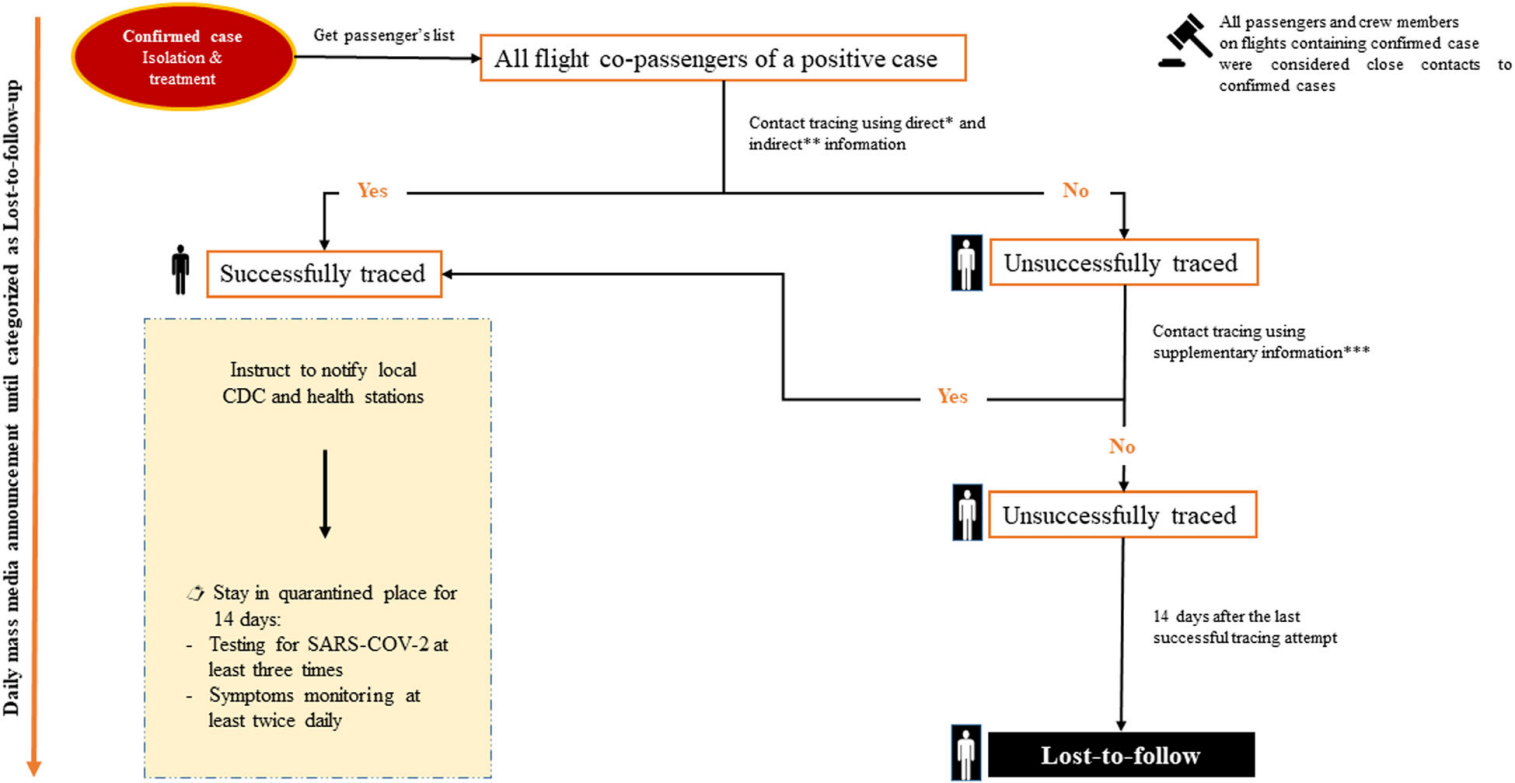
Procedure of contact tracing for flights with COVID-19 infected cases in Vietnam. ^*^ Direct contact information: email, telephone, social media accounts. ^**^ Indirect information: residential address, workplace address, tourism company. ^***^ Supplementary information: temporary/permanent residential registration at local authorities, police department records, tour registration at tourism bureaus, relevant embassies, and immigration bureau data

Contact tracing of flight passengers for COVID-19 in Vietnam during 2020 was a complex operation that involved multiple jurisdictions and institutions, including the Ministry of Health (MoH); the National Institute of Hygiene and Epidemiology (NIHE); the Rapid Response Team (RRT) of the National Steering Committee of COVID-19 Prevention and Control; the Civil Aviation Administration and Immigration Bureau; 63 provincial Centres for Disease Controls and Prevention (CDC); and the local police. The MoH assigned the NIHE as the key institution to manage and store data of contact tracing for flight passengers, including SARS-CoV-2 test results. Whenever a laboratory-confirmed polymerase chain reaction (PCR) test was positive, this was immediately reported by the testing facility to the NIHE. The NIHE then passed on the contact information of confirmed cases to the RRT. The RRT contacted and interviewed all COVID-19 cases for their travel history on international or domestic flights within 14 days from date of first symptom onset or date of first positive test result, whichever came first. The RRT then requested the Civil Aviation Administration and Immigration Bureau to provide flight manifests including passenger names, seat numbers, and personal contact details. The RRT shared this information with provincial CDCs and the local police. Local CDC staff and the local police jointly contacted passengers using email, telephone, or social media accounts if available. If necessary, other information such as residential or workplace addresses, information from tourism companies and embassies (for tourists), temporary/permanent residential registration data from local authorities, police department records, and immigration bureau data were also obtained. Furthermore, the MoH issued daily public announcements of flight details on mass media to advise all passengers on these flights to self-present immediately at designated facilities (Fig. 1).

All passengers on a flight with a positive case were traced, with their tracing status (tracing ongoing, successfully traced, transited out of the country, lost-to-follow-up) updated daily by local CDC staff on the national COVID-19 Contact Tracing Management System. All successfully traced passengers were instructed to immediately self-quarantine while awaiting health staff to take a nasopharyngeal swab for SARS-CoV-2 PCR testing. All traced passengers were subsequently transferred to designated quarantine areas regardless of their test result. Passengers who had already left Vietnam were instructed to contact respective health jurisdictions at their current location. Contact tracing of a flight was closed 14 days after the last successful tracing attempt occurred; all passengers not reached at that point were declared lost-to-follow-up. For this analysis, contact tracing completion was marked as the date when the last passenger on the flight was successfully traced.

#### Data collection

We extracted data for flight numbers, place of departure, arrival dates, total passenger numbers on each flight, passengers’ nationalities, the epidemiological index case detection method of each flight, date of first laboratory confirmation of the index case, date of contact tracing initiation and completion, and final contact tracing status (successfully traced; transited out of the country; lost-to-follow-up) from the national COVID-19 Contact Tracing Management System.

#### Statistical analysis

We used frequency statistics to describe the total number of passengers needed to be traced and successfully traced each day. We calculated time intervals between the date of index case confirmation and the date of contact tracing initiation (interval I), and the date of contact tracing initiation and date of completion (interval II), both overall and separately by method of index case detection (self-presentation vs. mandatory testing). The outcome of interest was the total duration of contact tracing, defined as time between date of index case confirmation and date of completion (interval I + II).

We calculated Kaplan Meier estimates to plot the probability of unsuccessful tracing (survival probability) over time and used log-rank tests to compare the cumulative hazards of successful tracing between both methods of index case detection (self-presentation after arrival or mandatory testing at arrival), passenger’s nationality (Vietnamese vs. non-Vietnamese), and number of passengers per flight requiring tracing (< 50, 50–99, > 100). If a passenger remained untraced at date of analysis (31 March 2020), the observation period was considered right censored. In addition, we developed a multivariable Poisson regression model to assess the association between index case detection method and timeliness of contact tracing adjusted for passenger nationality and the number of arriving passengers per plane. Data were analyzed using R version 4.0.2 [16].

## Results

**Fig. 2:**
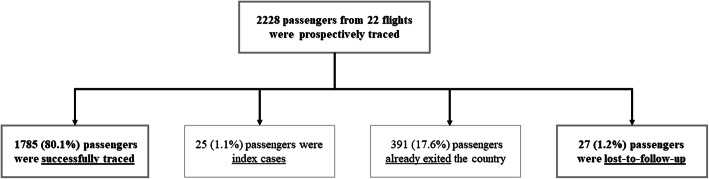
Contact tracing outcomes of 22 flights with infected COVID-19 cases during March 2020 in Vietnam

We included 22 flights arriving to Vietnam between 2 and 19 March 2020 in our analysis, with a total number of passengers of 2228; of these, 25 were index cases (1.1%). Most passengers were successfully traced (80.1%; *n* = 1785), while 1.2% (27 passengers) were lost-to-follow-up. The remaining 391 passengers (17.6%) had already transited to other countries at time of investigation (Fig. 2).

**Fig. 3:**
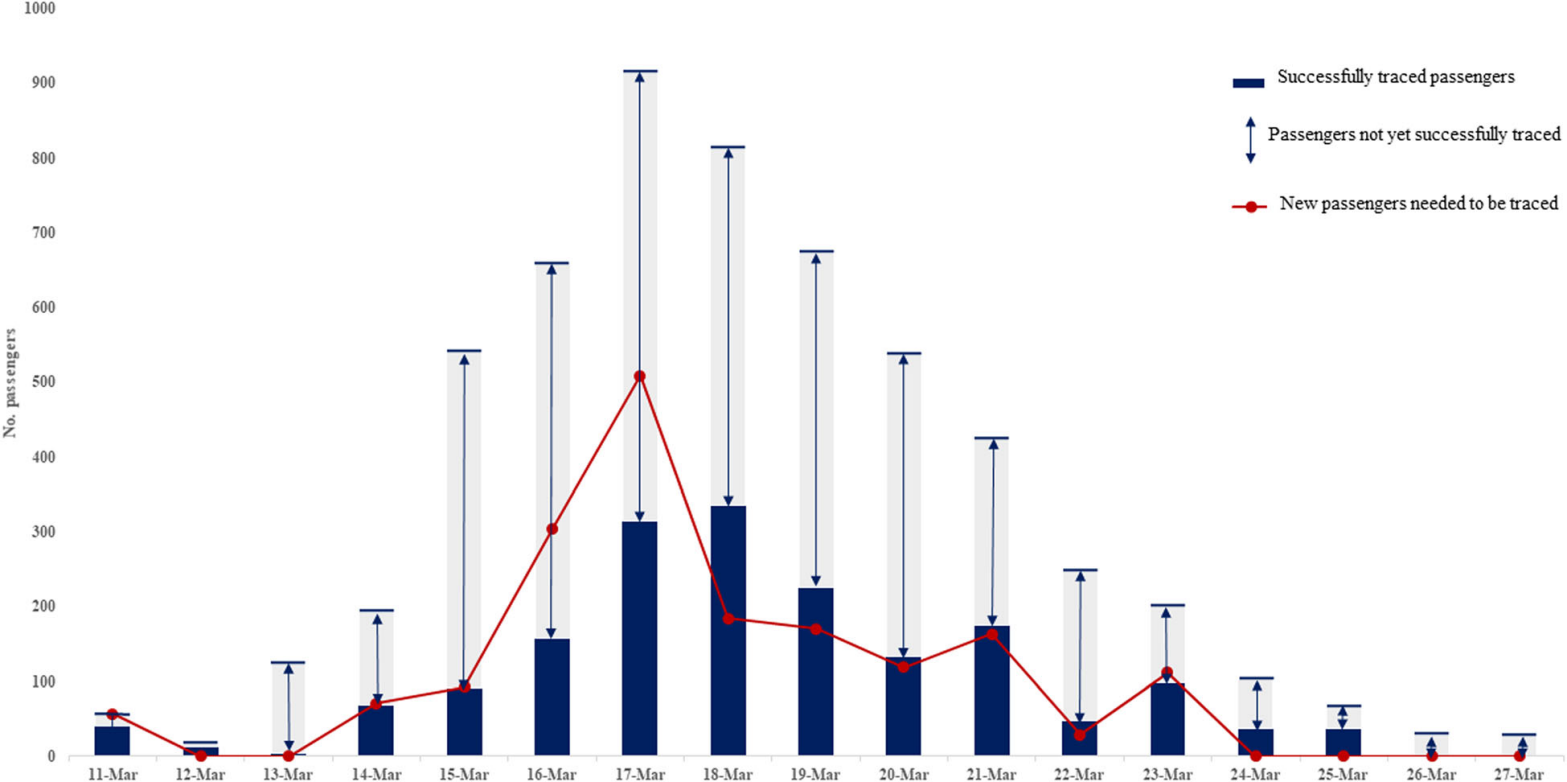
Number of passengers needed to be traced, successfully traced, and not yet successfully traced^*^ each day. ^*^Number of passengers not yet successfully traced = number of passengers needed to be traced – number of passengers successfully traced. Number of passengers needed to be traced at each day = number of passengers not yet succesfully traced from previous day(s) + new number of passenger needed to be traced. Note: We excluded index cases and transited passengers from this analysis since we did not attempt to trace them

Figure 3 presents the volume of 1812 passengers for which prospective contact tracing was done over time. The median number of passengers successfully traced was 69 (Interquartile range - IQR: 37–162) per day, while the daily median number of new passengers needed to be traced and the total number of passengers needed to be traced was 71 (IQR: 0–165) and 202 (IQR: 66–571), respectively. The total number of passengers requiring tracing was above 400 on each day between 15 March and 21 March. More than 50% of passengers remained untraced during these high workload days.

**Fig. 4:**
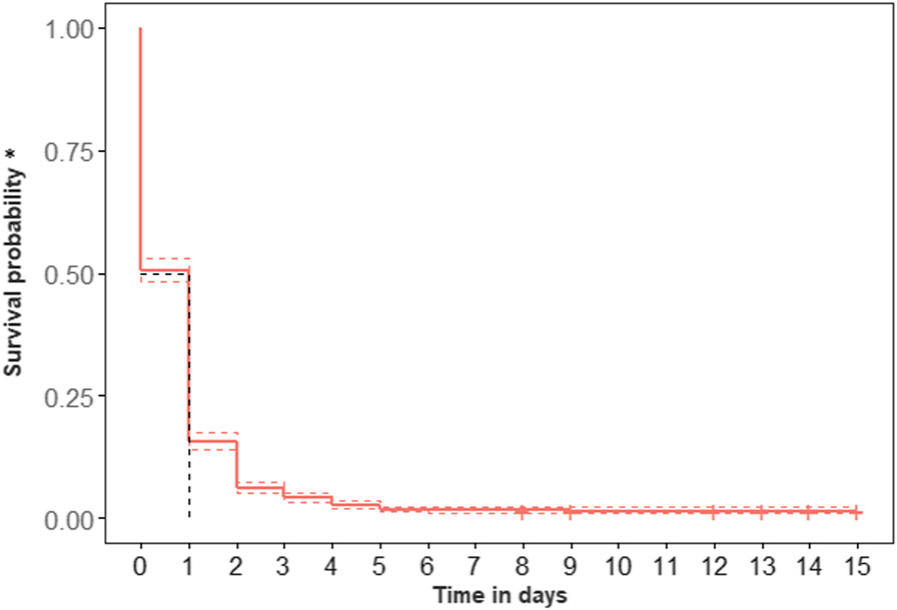
The cumulative probability of unsuccessful tracing for 1812 passengers on 22 flights during March 2020. * Survival probability was estimated using Kaplan Meier and interpreted as probability of successful tracing. Note: Dotted red lines indicated the 95% confidence intervals. The black dashed line indicates the time when half of all passengers are expected to be traced

The percentage of passengers needed to be traced reduced rapidly during the first 2 days of contact tracing (Fig. 4).

A total of 10 index cases from nine flights carrying 1050 passengers were detected by self-presentation, while 15 index cases from 13 flights carrying 1178 passengers were detected by mandatory testing at arrival. Details of the timeline and duration of contact tracing for all 22 traced flights are shown in Additional file 2. There was an overall median delay of 1 day (IQR: 1–2) between index case confirmation and initiation of contact tracing (Interval I), and an overall median delay of 3 days (IQR: 2–5) between initiation and completion of contact tracing process (Interval II). Interval II was longer for flights with an index case detected by self-presentation (median 5; IQR: 4–7) than for flights with an index case detected by mandatory SARS-CoV-2 testing (median 2; IQR: 2–3) (*p*-value = 0.005). Similarly, the combined time interval between index case confirmation and contact tracing completion for flights with index case detected by self-presentation (median = 7; IQR: 5–8) was longer than for flights with an index case detected by mandatory testing (median = 4; IQR: 3–5) (*p*-value 0.018) (Table 1).

**Table 1:** Time interval of contact tracing of passengers on 22 flights arriving Vietnam from 6 March to 21 March by methods of index case detection

Time	Total (***n*** = 22) *Median (IQR)*	Method of index case detection *Median (IQR)*		***p***-value^a^
		Self-presentation (***n*** = 9)	Mandatory testing (***n*** = 13)	
Interval I	1 (1–2)	1 (1–2)	2 (1–2)	0.34
Interval II	3 (2–5)	5 (4–7)	2 (2–3)	0.005
Total time	4.5 (4–6)	7 (5–8)	4 (3–5)	0.018

Note: Interval I is defined as time intervals between date of index case confirmation to date of contact tracing initiation; Interval II is time intervals between date of contact tracing initiation to completion; Total time = Interval I + Interval II

^a^Interval I, Interval II, and Total time by method of index case detection were compared using Wilcoxon rank-sum tests

**Fig. 5:**
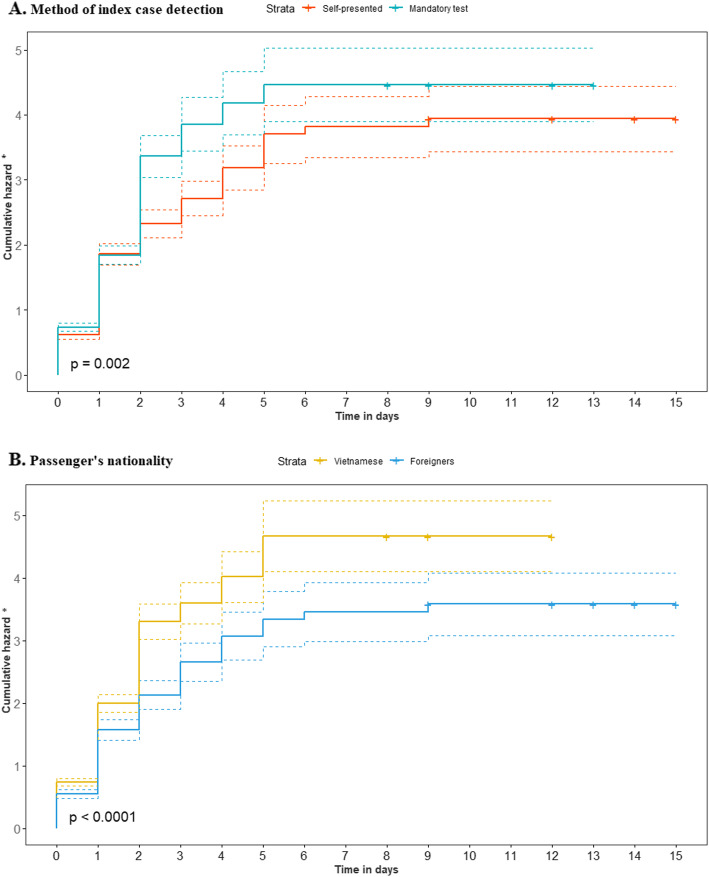
Cumulative hazard of successful tracing for passengers by method of index case detection (**a**) and nationality of passengers (**b**). * Cumulative hazards were estimated using Kaplan Meier and interpreted as probability of successful trace. *p*-value was calculated from log-rank tests. Note: Dotted lines indicated the 95% confidence intervals

Figure 5 illustrates the cumulative hazards of successful tracing over time by method of index case detection, the passenger’s nationality, and the number of arriving passengers per plane. Cumulative hazards were higher for passengers on flights with an index case detected by mandatory testing than for passengers on flights with index cases detected by self-presentation (*p*-value = 0.002) and for Vietnamese passengers than for non-Vietnamese nationals (*p*-value< 0.001).

Table 2 shows univariate and multivariable analyses assessing the association between method of index case detection and timeliness of contact tracing. The duration of contact tracing was longer for people arriving on flights with large passenger volumes, with an Incidence Rate Ratio (IRR) of 1.36 (95% confidence interval (95%CI): 0.62–2.98) and 1.67 (95%CI: 0.75–3.73) for passengers on flights with 50–99 passengers and with more than 100 passengers, respectively, compared to flights with small volumes (below 50 passengers). After controlling for nationality and passenger volume per plane, the contact tracing duration for passengers where the index case was detected by self-presentation was longer than for passengers on flights where the index case was detected by mandatory testing upon arrival (IRR: 1.94, 95%CI: 1.17–3.21).

**Table 2:** Relationship between method of index case detection and timeliness^a^ of contact tracing for passengers on 22 flights arriving in Vietnam from 6 March to 21 March

	***n*** (%)	Univariate Associations		Model 1		Model 2		Model 3	
		IRR	95%CI	IRR	95%CI	IRR	95%CI	IRR	95%CI
**Method of index case detection**									
Mandatory testing	9 (40.9)	**Ref**	**–**	**Ref**	**–**	**Ref**	**–**	**Ref**	**–**
Self-presentation	13 (59.1)	**1.61**	**1.11–2.34**	**1.86**	**1.18–2.92**	**1.59**	**1.07–2.34**	**1.94**	**1.17–3.21**
**Percentage of foreigners per flight**									
≤10%	7 (31.8)	Ref	–	Ref	–			Ref	–
10.1–20%	4 (18.2)	0.9	0.51–1.59	1.12	0.61–2.04			1.15	0.61–2.14
20.1–30%	4 (18.2)	1.1	0.65–1.87	0.96	0.56–1.65			0.89	0.51–1.56
> 30%	7 (31.8)	1	0.63–1.60	0.77	0.47–1.28			0.74	0.44–1.24
**Number of passengers per flight**^b^									
< 50	2 (9.1)	Ref	–			Ref	–	Ref	–
50–99	13 (59.1)	1.36	0.62–2.98			1.07	0.48–2.42	0.91	0.38–2.17
100+	7 (31.8)	1.67	0.75–3.73			1.34	0.58–3.06	1.18	0.49–2.83

Model 1: Timeliness by method of index case detection adjusted for percentage of foreigners per flight

Model 2: Timeliness by method of index case detection adjusted for number of passengers per flight

Model 3: Timeliness by method of index case detection adjusted for percentage of foreigners per flight and number of passengers per flight

^a^ Total time between date of index case confirmation to contact tracing completion (Interval I + II)

^b^ Total number of passengers per flight for who contact tracing was performed

## Discussion

We evaluated the timeliness of contact tracing and factors associated with timeliness among 22 flights between 2 March and 19 March 2020, shortly before Vietnam’s government issued the universal test and quarantine policy for all inbound passengers. Our findings suggest that mandatory SARS-CoV-2 testing at arrival can help to reduce the contact tracing duration of passengers upon detection of an index case.

This study provides important evidence on integrated contact tracing of flight passengers in a low-middle income country in the midst of the ongoing COVID-19 pandemic.

The short tracing duration for passengers on flights with index case detection by mandatory testing at the time of arrival can be explained with the reduced delay in index case detection compared to flights where index cases self-presented, which in turn meant that passengers on these flights had less time to disperse throughout the country and change locations multiple times, which incurred delays. However, this test and quarantine policy by area of departure individually for each passenger (i.e., passengers on the same flight could leave the airport without testing or quarantine if they did not depart from a designated high-risk area and would only be traced if a passenger on the same flight tested positive) was complex to implement. With SARS-CoV-2 increasingly spreading to more and more countries in March 2020 anyways, it was soon replaced by a blanket mandatory quarantine policy for all incoming passengers irrespective of origin.

Although most passengers were successfully traced within 2 days of initiating contact tracing, we still recorded a long tail of unreached passengers over time, indicating a lengthy process to locate some passengers. The reasons for this were manifold and, though reasons for unsuccessful tracing were not recorded systematically, several of them seem to have affected international tourists more than Vietnamese nationals, which might explain the lower cumulative hazards of successful tracing in non-Vietnamese than in Vietnamese nationals. For example, fast-changing locations, lack of local mobile phone contact details, and language barriers were obstacles often experienced by the contact tracers according to our anecdotal experience. Some of these passengers were successfully reached via email, their social media accounts, or tourism companies, while for others the involvement of their embassies or the Vietnamese immigration bureau was required, which added to the delay. Although the MoH issued information of flights with confirmed cases on mass media in both Vietnamese and English, this might not have reached all international tourists, which can explain further delays in this group. Incorrect or missing contact information added complexity and challenges to trace passengers. All inbound passengers from international flights had to complete a health declaration form either via a paper-based or web-based version, which was not always done correctly and completely. While the web-based data was directly linked to the passengers’ management system, paper-based forms had to be entered manually by the Civil Aviation Administration and Immigration Bureau. Due to the large daily volume of paper-based records at that time, data entry errors might have occurred more frequently during high workload days.

Our results suggest that contact tracing was more time-consuming with increasing passenger volume of that flights, which makes sense in light of the sudden overload of the system through large volume flights. Furthermore, all flights with medium and high volumes were international connections carrying large numbers of non-Vietnamese nationals. Collecting passenger details from foreign airlines also took longer because of differences in time zones and the need to convince the airline companies to hand over passenger lists, which often required to issue official request letters.

Challenged by a surge of passengers needed to be traced, the contact tracing process in Vietnam adapt, as shown by the increasing number of passengers successfully traced. However, this adaptation did not seem to have been enough during days with a high workload, since more than half of the passengers needed to be traced were not reached during these days. While contact tracing was a crucial component for preventing the spread of COVID-19 in the community [17], it is important to integrate contact tracing with other public health interventions such as border control and arrival testing. The high number of imported cases due to international travel has prevailed over time in many countries leading to overstretched contact tracing capacities [18, 19]. With the early recognition of this possible challenge, the Vietnamese government decided on 21 March 2020 to mandatorily test and quarantine all inbound passengers to reduce the workload for contact tracing, even though only 94 confirmed cases had been recorded at that time [20].

We acknowledge several limitations of this study. Firstly, available data were recorded in days instead of hours, which adversely impacted the accuracy of our estimates. Secondly, for flights with index cases detected by mandatory testing, some passengers were already tested and quarantined upon arrival at the same time as the index cases. However, we could not exclude such passengers from our analysis due to data unavailability but we estimate the number to be relatively low since the list of designed high-risk areas, which required direct quarantine upon arrival only of those passengers who started their journey from a high-risk area but not of all passengers on that plane, was only gradually expanded (see Additional file 1). Since we treated such contacts as successfully traced at the first day of contact tracing initiation, any effect of this would have contributed to an underestimation of the observed difference in timeliness between the two methods of index case detection. Thirdly, our analysis did not explore contextual factors that may have impacted the contact tracing duration and contact tracing evolution over time such as overall daily capacity of the contact tracing system, organizational structure, administrative delays, information distribution, number of jurisdictions, availability of human resources, and skill levels of contact tracers. Future mixed-methods research is needed to answer these questions. Fourthly, no data on the causes of delay or loss to follow up was systematically recorded and were thus not available for our analysis, which would have enriched the interpretation of our findings.

## Conclusion

Contact tracing among flight passengers in the early stage of the COVID-19 epidemic in Vietnam was timely, although delays occurred on high workload days. Mandatory SARS-CoV-2 testing at arrival may reduce contact tracing duration and should be considered as an integrated screening tool for flight passengers from high-risk areas when entering low-transmission countries with limited contact tracing capacity. We recommend a standardized risk-based contact tracing approach for flight passengers during the ongoing COVID-19 epidemic.

## Supplementary Information

**Additional file 1.** Public health interventions to prevent transmission from flights in Vietnam.

**Additional file 2.** Timetable from arrival to contact tracing completion for 22 tracing flights, March 2020, Vietnam.

## Data Availability

The datasets used and/or analyzed during the current study are available from the corresponding author (Ha-Linh Quach) or the first author (Ngoc-Anh Hoang) on reasonable request.

## Abbreviations

RRT: Rapid Response Team
NIHE: National Institute of Hygiene and Epidemiology
PCR: Polymerase chain reaction
CDC: Centres for Disease Controls and Prevention
MoH: Ministry of Health
IRR: Incidence Rate Ratio
IQR: Interquartile range
