# Supplementary material for: Timeliness of contact tracing among flight passengers during the COVID-19 epidemic in Vietnam
Source: BMC Infect Dis. 2021 Apr 28;21:393. doi: 10.1186/s12879-021-06067-x (PMC8080478; doi:10.1186/s12879-021-06067-x)
Supplement: Supplementary file 1 — Additional file 1. Public health interventions to prevent transmission from flights in Vietnam. [file 12879_2021_6067_MOESM1_ESM.docx]

**Additional file 1. Public health interventions to prevent transmission from flights in Vietnam**

| **Starting date** | **Actions** |
| --- | --- |
| 1 January | Temperature screening^*^ for passengers from Hubei, China at entry points in all borders (airport, sea, land) in Vietnam |
| 6 March | Case finding and contact tracing for all passengers on flights with identified infected case(s).  Mandatory health declaration for all inbound passengers from international flights |
| 14 March | SARS-CoV-2 testing at arrival and quarantine for passengers from the United Kingdom and 26 Schengen countries |
| 18 March | Expanded SARS-CoV-2 testing at arrival and quarantine for passengers from the United States, selected Southeast Asian countries^✝^, and Russia |
| 21 March | SARS-CoV-2 testing at arrival and quarantine for all passengers from international flights regardless of place of departure |
| 28 March | All international flights halted |

*^*^ Including both forehead temperature measurement and heat image*

*^✝^ Brunei, Cambodia, Indonesia, Laos, Malaysia, Myanmar, the Philippines, Singapore, Thailand*
