# Supplementary figures and images for: Timeliness of contact tracing among flight passengers during the COVID-19 epidemic in Vietnam
Source: BMC Infect Dis. 2021 Apr 28;21:393. doi: 10.1186/s12879-021-06067-x (PMC8080478; doi:10.1186/s12879-021-06067-x)

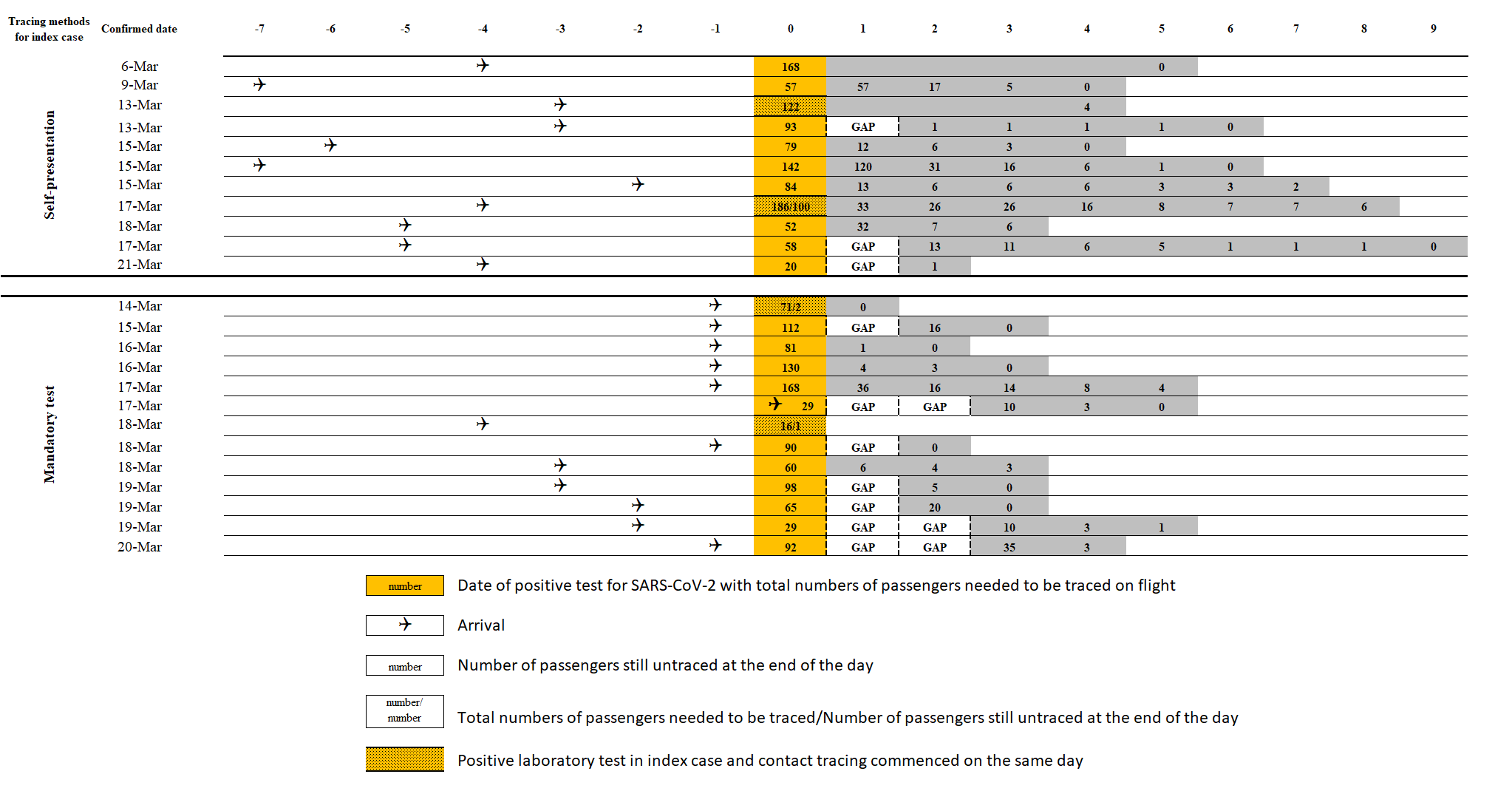

Supplement: Supplementary file 2 — Additional file 2. Timetable from arrival to contact tracing completion for 22 tracing flights, March 2020, Vietnam. [file 12879_2021_6067_MOESM2_ESM.png]
